# Verifying Feighner’s Hypothesis; Anorexia Nervosa Is Not a Psychiatric Disorder

**DOI:** 10.3389/fpsyg.2019.02110

**Published:** 2019-09-16

**Authors:** Per Södersten, Ulf Brodin, Modjtaba Zandian, Cecilia E. K. Bergh

**Affiliations:** Karolinska Institutet, Mandometer Clinics, Huddinge, Sweden

**Keywords:** anorexia nervosa, mental causation, evolution, starvation, psychiatric diagnosis, treatment

## Abstract

Mental causation takes explanatory priority over evolutionary biology in most accounts of eating disorders. The evolutionary threat of starvation has produced a brain that assists us in the search for food and mental change emerges as a consequence. The major mental causation hypothesis: anxiety causes eating disorders, has been extensively tested and falsified. The subsidiary hypothesis: anxiety and eating disorders are caused by the same genotype, generates inconsistent results because the phenotypes are not traits, but vary along dimensions. Challenging the mental causation hypothesis in Feighner et al. (1972) noted that anorexic patients are physically hyperactive, hoarding for food, and they are rewarded for maintaining a low body weight. In 1996, Feighner’s hypothesis was formalized, relating the patients’ behavioral phenotype to the brain mechanisms of reward and attention (Bergh and Södersten, 1996), and in 2002, the hypothesis was clinically verified by training patients how to eat normally, thus improving outcomes (Bergh et al., 2002). Seventeen years later we provide evidence supporting Feighner’s hypothesis by demonstrating that in 2012, 20 out of 37 patients who were referred by a psychiatrist, had a psychiatric diagnosis that differed from the diagnosis indicated by the SCID-I. Out of the 174 patients who were admitted in 2012, most through self-referral, there was significant disagreement between the outcomes of the SCID-I interview and the patient’s subjective experience of a psychiatric problem in 110 of the cases. In addition, 358 anorexic patients treated to remission scored high on the Comprehensive Psychopathological Rating Scale, but an item response analysis indicated one (unknown) underlying dimension, rather than the three dimensions the scale can dissociate in patients with psychiatric disorders. These results indicate that psychiatric diagnoses, which are reliable and valid in patients with psychiatric disorders, are less well suited for patients with anorexia. The results are in accord with the hypothesis of the present Research Topic, that eating disorders are not always caused by disturbed psychological processes, and support the alternative, clinically relevant hypothesis that the behavioral phenotype of the patients should be addressed directly.

## Introduction

*Scientific advances often come from uncovering a hitherto unseen aspect of things as a result, not so much of using some new instrument, but rather of looking at objects from a different angle* (Jacob, 1977)

A large population-based twin study in Finland reported that most women diagnosed with anorexia nervosa recovered clinically within 5 years and thereafter progressed toward full recovery (Keski-Rahkonen et al., 2007). This interesting study should be revisited, because several reviews of the outcome in anorexia nervosa have concluded the opposite, i.e., although patients may experience symptomatic relief in the short term, remission is uncommon, many patients relapse within a year of discharge, and outcome in the longer term is poor or unknown (Eckert et al., 1995; Ben-Tovim et al., 2001; Zipfel et al., 2015; Södersten et al., 2017; Fisher et al., 2018; Zeeck et al., 2018). Hence, the purpose of this Research Topic, to break the impasse, is appropriate. As the introductory quote from Francois Jacob suggests, it may be useful to view anorexia from a different angle rather than relying on the hypothetical cause-effect relationships that underlie standards of care.

A possible explanation for the limited success of presently used standard treatments was provided 47 years ago when Feighner et al. suggested that anorexia is not caused by a mental disorder (Feighner et al., 1972), questioning the assumption that underlies standards of care (Zipfel et al., 2015; Zeeck et al., 2018). The mental causation hypothesis is expressed in a position paper from the Academy of for Eating Disorders: “eating disorders are … serious mental illnesses … caused by neurobiological disorder(s) of the brain … Anxiety disorders often predate the onset of [anorexia nervosa]” (Klump et al., 2009). It seems likely that challenging the mental causation hypothesis made Feighner’s hypothesis a “sleeping beauty,” i.e., “a paper whose importance is not recognized for several years after publication” (Ke et al., 2015). We suggest that it is time to wake up Feighner’s hypothesis.

This report will present an updated biological theory of the relationship among the mind, starvation and the brain. Outcomes of tests of the mental causation hypothesis are reviewed and original clinical data are presented that support Feighner’s hypothesis. A 23 years old framework for an improved treatment is then re-launched, including a hypothesis on how the brain mediates among eating behavior and mental symptoms.

## Biological Theory

*Nothing in biology makes sense except in the light of evolution* (Dobzhansky, 1973)

Forty years after Dobzhansky stated the importance of evolution for understanding biology, most scientists agreed, but this perspective has been neglected outside the biological sciences (Striedter et al., 2012). And most accounts of anorexia nervosa still do not consider the evolutionary significance of starvation. This is surprising because evolutionary psychology and psychiatry are now long established fields (Varga, 2012; Nesse, 2019).

### Evolution and the Mind

Humans offer mental causes, including intentionality and agency, to explain purposeful behavior (Bloom, 2008, 2012), i.e., “In many populations with Western European roots, the prevailing belief is that minds and mental states cause behaviors” (McNamara et al., 2019). Over the course of evolution, the psychological process of mental causation, i.e., dualism, developed in parallel with the cognitive systems that are necessary for understanding of the physical properties of our environment (Bloom, 2012). At the psychological level, the idea that everything has a physical basis [for a discussion of physicalism see Stoljar (2017)] normally co-exists with dualism [for a discussion of dualism see Robinson (2017)], but the two are separable, distinct processes (Bloom, 2012).

Bloom suggested that “we are born dualists. we naturally believe in an immaterial soul, in spirits …,” and significantly that we are dualists “for clear adaptive reasons” (Bloom, 2012). Earlier, Darwin raised this possibility by suggesting that “mental powers” emerged over the course of evolution (Jacyna, 2009). Now it has been demonstrated that these dualistic psychological processes have played a significant role in between-group competition and in within-group co-operation and thus in the emergence of shared systems of belief, such as religions; that is to say, dualism and mental causation have facilitated cultural evolution (Norenzayan et al., 2016; Purzycki et al., 2016; Lang et al., 2019). Interestingly, psychological processes of mental causation can emerge after within-group co-operation and engage neural substrates that can be dissociated from the neural substrates engaged in analytical thinking, the two are in competition (Jack et al., 2016; Whitehouse et al., 2019).

However, mental causation is a feeling or an illusion, rather than an explanation; one is reminded of the behaviorist comment: “we talk about free will and choice when we know about behavior but not its cause” (Skinner, 1977; Wegner and Wheatley, 1999; Wegner, 2003, 2004, 2018). Yet, systems of belief, including false beliefs, are often resistant to change even in the face of contradictory scientific evidence, and retrospective examination of the “facts” may be ineffective and can even strengthen the false belief (Lewandowsky et al., 2012; Fazio et al., 2015; Yousif et al., 2019). Today, research on “fake news” has disclosed some of the factors involved, including political and economic interests, denialist campaigns, shared values, maintenance of social networks and so forth (Jost et al., 2018; Lewandowsky et al., 2019). Nevertheless critical examination of the available data is essential for progress in science, for the translation of science into clinical practice, and for guiding public health policies (Jacob, 1977).

### Evolution and Starvation, a Brain Designed for the Search for Food

Eating and the search for food have dominated human life in feast and famine alike (Södersten et al., 2008). Using an animal model, Epling and Pierce related the increased physical activity that emerges in starvation and the high physical activity of anorexic patients to the search of food (Epling and Pierce, 1984, 1988). An extensive literature on the evolutionary importance of foraging had accumulated at the time (Stephens and Krebs, 1986). This suggestion was novel because it departed from the mental causation hypothesis as recognized by Guisinger (2003), and today evidence has accumulated that an evolutionary perspective can inform our understanding of anorexia as suggested by Epling and Pierce. Thus, the neural pathways that are activated by the shortage of food assist animals in searching for food, rather than eating, in fact foraging for food can decrease food intake (Ammar et al., 2000; Södersten et al., 2008; Schneider et al., 2013). The neuroendocrine cells in the hypothalamic arcuate nucleus that are activated by starvation are involved in “shaping (the) behavioral choices” needed for foraging, and these behaviors outcompete most other behavioral choices (Chen et al., 2015; Dietrich et al., 2015; Burnett et al., 2016; Södersten et al., 2019b). The importance of this behavioral shift is obvious, as starving animals and humans must find food. The role of the brain is permissive, not causal (Södersten et al., 2011).

### Anorexia Nervosa and the Mind-Body Problem

The observation that reduced food intake provokes increased physical activity was made a hundred years ago in experimental psychology (Richter, 1922). Shortly thereafter, it was demonstrated experimentally that food intake decreases as a consequence of an increase in physical activity (Skinner, 1938). A comprehensive overview of activity-based anorexia, the animal model for anorexia nervosa that emerged from these initial observations was published more recently (Gutierrez, 2013). The importance of the model for the present discussion of mental causation lies in the reports of the mental effects in healthy humans who participated in a starvation experiment (Keys et al., 1950). While physical activity was an experimental variable rather than a measure of outcome in that study, the increase in physical activity in the search for food has been documented in hundreds of thousands of people during enforced starvation (Södersten et al., 2008).

The humans in the starvation experiment were interviewed not long ago and the mental effects they experienced were recently published (Kalm and Semba, 2005; Tucker, 2007; Case, 2018; Eckert et al., 2018; University of Minnesota, 2018). While “food became an obsession for the participants” (Kalm and Semba, 2005), the mental effects came as a surprise:

*What I wasn’t expecting was the effect it would have on the mind; the total feeling of, I guess, depression, the total occupation with the idea of food* (participant)

Crow interviewed 19 of the 36 subjects long after the experiment and noted that they had all of the mental symptoms that are characteristic of anorexics:

*The similarity between these* [*effects of starvation*] *and what’s seen in clinical settings treating people with eating disorders is really quite striking. I knew all about this stuff before I really knew very much about what happened in the study just from working on an eating disorders unit* (Crow, 2004)

It is also the case that the mental/behavioral effects of starvation are reversible with appropriate re-feeding (Eckert et al., 2018).

### Conclusion

The brain mediates eating-behavior responses to food restriction and has evolved to assist the individual in the search for food. Moreover, healthy humans develop the mental symptoms of anorexia nervosa during food restriction because they are starving, but when the same response is seen in anorexia, humans assume a mental causation of those symptoms by default, rather than assuming that the food restriction is causing that response.

## The Mental Causation Hypothesis

*Anorexia nervosa is a graphic illustration of the influence of emotions on bodily functions* (DuBois, 1949)

Mental factors have long been thought to cause anorexia, rather than thinking that food deprivation causes the mental problems. Given the complexities of comprehending the concept of whether one of these two factors is causative, it is helpful to analyze the concept of co-morbidity.

### Co-morbidity

It is not clear what is cause and what is effect among mental symptoms and the eating disorder and much of the problem with understanding this issue concerns the concept of co-morbidity. However, the analysis of causation is complex (Hacker, 2010) and there are at least two problems.

First, the original idea of co-morbidity was of co-existing, but independent, disorders (Feinstein, 1970). From this perspective, co-existing psychiatric symptoms and disordered eating behaviors are not causally related. Yet, the concept of co-morbidity has led to the clustering of psychiatric symptoms that are not independent (Maj, 2005), and the mental causation hypothesis assumes that they are related.

Second, mental disorders are thought to be “risk factors” for anorexia. Two definitions of risk are useful: “the *probability* of an unwanted event which may or may not occur” and the “*cause* of an unwanted event which may or may not occur” (Hansson, 2018). Regarding the first definition, one needs to determine why a mental disorder increases the probability of experiencing an eating disorder. There is limited, if any, information that clarifies this matter [see discussion in Södersten et al. (2006)]. We therefore will use the second definition, i.e., risk = cause, to examine the evidence for and against the hypothesis that a mental disorder causes anorexia nervosa, the prototypical eating disorder.

We avoid the distinction between cause, mediator, mechanism, and modulator in psychotherapeutic research (Kazdin, 2007; Tryon, 2018). In any analysis, cause differs from mechanism and mediators and modulators are replaced by mechanisms as research progresses.

### The OCD Hypothesis

*Childhood anxiety represents one important genetically mediated pathway toward the development of anorexia nervosa*… (Kaye et al., 2004)

Anorexia was once thought to be a symptom of anxiety, rather than a separate disorder [reviewed in Wu (2008)], but it is now thought that an anxiety disorder, specifically obsessive compulsive disorder (OCD), causes anorexia. We will examine the attempts at verifying this version of the mental causation hypothesis, because it uncovers some problems related to the methods used to assess this hypothesis.

#### Self-Reported OCD in Anorexia Nervosa

Retrospective interviews were first used to examine the possibility that anxiety disorders cause anorexia nervosa. In support of that hypothesis, 60% of 68 women with anorexia nervosa recalled having an anxiety disorder, and 90% were thought to have had both childhood overanxious disorder and social anxiety disorder before they had anorexia nervosa (Bulik et al., 1997). While these anxiety disorders were considered to be non-specific risk factors for psychopathology later in life, it was suggested that OCD is a specific risk factor for anorexia nervosa, although the presence of OCD before anorexia nervosa was not reported (Bulik et al., 1997).

In another study, 42% of 672 patients with eating disorders reported one or more anxiety disorders in childhood and 23% said that they had OCD before they developed anorexia nervosa (Kaye et al., 2004). But re-calculation of the data in their study showed that 7% of the patients had OCD before they had anorexia nervosa (Södersten and Bergh, 2006). Upon commenting on the re-calculation, the authors stated that at least 14% of the patients had OCD before they had anorexia nervosa and that 36% of the patients in an unpublished study had an anxiety disorder before they had anorexia nervosa (Kaye et al., 2006). They then reported that 39.1% of 647 patients with anorexia nervosa had an anxiety disorders and that 94.4% of the patients had the anxiety disorder before they developed anorexia (Raney et al., 2008). But rather than having OCD, that anxiety disorder that preceded anorexia was childhood overanxious disorder, the disorder that previously was thought to be a non-specific risk factor for psychopathologies in adulthood (Bulik et al., 1997). It was also pointed out that childhood overanxious disorder is no longer a recognized psychiatric disorder (Raney et al., 2008) and the hypothesis that it predates anorexia was subsequently falsified (Buckner et al., 2010).

These discrepancies led others to conclude that the OCD hypothesis was not supported by the existing data (Godart et al., 2006; Swinbourne and Touyz, 2007). It was also pointed out that the causal relationship among anxiety and eating disorders might be the opposite, i.e., that anorexia (or starvation) causes anxiety (Micali et al., 2015). This hypothesis was verified by Keys’ study on starvation (O’Brien and Vincent, 2003). Yet, a recent review re-affirmed that anxiety disorders “typically” precede anorexia nervosa (Zipfel et al., 2015).

#### Registry Studies That Assess the Role of OCD in Eating Disorders

Recalled disorders produce unreliable estimates of the actual experiences in epidemiological studies (Regier et al., 1984), and it should be obvious that self-reported retrospective data make it difficult “to disentangle causes from effect” (Brander et al., 2016). However, the existence of large national data registries in which the dates of onset for different diagnoses are available allows for a more accurate examination of the putative role of OCD in the development of anorexia.

According to the introductory literature review of the first longitudinal registry study as many as 83% of patients with anorexia nervosa can be diagnosed with anxiety disorders, which precede the onset of anorexia nervosa “in most patients” (Meier et al., 2015). But that study subsequently found that 3.5% of 5,065 patients had actually been diagnosed with a specific anxiety disorder before they were diagnosed with anorexia nervosa, and only 1.9% had been diagnosed with OCD before developing anorexia nervosa (Meier et al., 2015). Hence, OCD did not predate anorexia in 98.1% of the patients, precluding the possibility that it was causal in the development of anorexia nervosa. It should also be noted how much lower the documented diagnoses of anxiety and OCD are compared to the self-reported estimates of these disorders.

Despite the evidence, the OCD hypothesis has been maintained by reporting increases in the risk for anorexia among patients diagnosed with OCD compared to healthy subjects in a national registry study. Thus, 0.7% out of 19,069 men and women with OCD had developed anorexia nervosa compared with 0.2% out of 190,690 healthy men and women (Cederlöf et al., 2015). While the relative risk of OCD preceding anorexia was 3.6 times that of those without OCD, it remains the case that 99.3% of OCD patients did not develop anorexia nervosa.

In the same study, 0.6% out of 19,512 men who had an OCD diagnosis also had an anorexia nervosa diagnosis, compared to 0.02% out of 195,120 healthy men. The relative risk of having anorexia nervosa among men with OCD had increased 37 times (Cederlöf et al., 2015). Rather than verifying the hypothesis that OCD causes anorexia, the results actually supported the hypothesis that anorexia causes OCD (O’Brien and Vincent, 2003), because anorexia nervosa before OCD was several-fold more common that OCD before anorexia, although both cause-effect relationships were uncommon (Cederlöf et al., 2015).

But the increased risk is more apparent than real. To illustrate the problem with these calculations: the probability of winning by drawing a number in a lottery is approximately equal to 0. Drawing ten numbers increases the probability of winning ten times, but the probability remains approximately equal to 0.

Conclusions regarding the increased risk for developing an eating disorder following OCD are further confounded by “… the possibility that subtle eating disorder symptoms were overlooked at initial assessment [of OCD]” (Cederlöf et al., 2015), as had been demonstrated in another longitudinal study (Micali et al., 2011). Also, OCD is a heterogeneous disorder in children (Zijlmans et al., 2017), and diagnosis is not reliable (Mataix-Cols et al., 2007; Rück et al., 2015). The recent suggestion that both patients with anorexia and patients with OCD don’t like to make mistakes (Levinson et al., 2018) stands out as a strained attempt at rescuing the OCD hypothesis in the face of accumulating evidence that argue against the hypothesis. Facing the absence of support from a study on approximately 14,000 children, the OCD hypothesis surrendered just a few months ago, although it was replaced by the equally uncertain hypotheses that childhood overanxious disorder or social anxiety cause anorexia (Kerr-Gaffney et al., 2018; Schaumberg et al., 2019).

#### Genetics and the OCD Hypothesis

While the history of the heredity of psychiatric disorders goes back several hundred years, the scientific framework for understanding it has remained the same, namely that hereditary factors predispose the individual to mental problems and that environmental factors subsequently cause the disorder (Porter, 2018). This is undoubtedly correct, a pioneer of behavioral genetics pointed out long ago that all behavior is the result of an interaction among genes and environment (Benzer, 1971).

Thus, the OCD hypothesis included the subsidiary hypothesis that OCD and anorexia have the same genetic basis (Kaye et al., 2004). However, it was reported that only 0.7% of the patients with OCD develop anorexia nervosa and that “the majority of genetic variance is disorder-specific” (Cederlöf et al., 2015). Paradoxically, a highly significant genetic risk factor for both OCD and anorexia nervosa was then reported in genome-wide-association studies (Brainstorm Consortium, 2018; Yilmaz et al., 2018; Watson et al., 2019). Thus, the subsidiary genetic OCD hypothesis was first falsified and then supported. These contrasting findings may be related to the use of registry data some of the studies versus data from genome-wide association data in the other studies. Note, however, that genome-wide association does not address the question of causation (Pearl, 2019).

#### Why the OCD Hypothesis Remains Elusive

One reason why tests of the OCD hypothesis have yielded inconsistent results may be because anxiety disorders as well as eating disorders are not distinct categories but vary along overlapping dimensions (Russell, 1979; Bergh et al., 2002; Fairburn et al., 2003; Wu, 2008). For example, an anxious person may have problems with sleep, attention, irritability, concentration, muscle tension, continual arousal, generalized worry, or fear. Thus, a multiple regression analysis using specific anxiety dimensions such as these revealed that OCD did not offer better discriminant validity than “general distress” in predicting eating disorders symptom than symptoms of panic and depression, which have overlapping symptoms with anxiety, questioning a specific OCD-eating disorders relationship (Wu, 2008).

Because attempts at classifying mental disorders into categories based on Mendelian genetics many years ago were unsuccessful, the existence of these categories was questioned (Porter, 2018). This issue was resolved 100 years ago when it was pointed out that with many genes involved, a continuous, normally distributed phenotype should be expected in the population (Fisher, 1918). Recent analyses confirm the involvement of many genes, perhaps the entire genome, in complex traits, including mental disorders (Boyle et al., 2017; Willsey et al., 2018; Wray et al., 2018), confirming the suggestion that psychiatric disorders vary along various symptomatic dimensions (Wu, 2008; Brainstorm Consortium, 2018).

Recent research offers mechanistic explanations for the failure to relate genotype to eating disorders diagnoses. It is long known that cells of different genotypes make up mosaics that constitute the basis for display of behavior (Hotta and Benzer, 1972). Mosaicism was recently demonstrated within brain neurons engaged in the ontogeny and display of behavior (Paquola et al., 2017). This opens for a vast number of gene-neurobiology-environment interactions. Add that behavioral phenotypes can be mediated by different neural networks (Nusbaum et al., 2017), and it will be difficult to verify the subsidiary OCD hypothesis.

### The ADHD Hypothesis

… *it is possible that symptoms of ADHD directly cause* [*eating disorder*] *behaviors* (Levin and Rawana, 2016)

While the OCD hypothesis has not been tested experimentally, another version of the mental causation hypothesis, the attention deficit hyperactivity disorder (ADHD) hypothesis, has been tested experimentally. Some have argued that research on the ADHD hypothesis has just started (Kaisari et al., 2017), whereas others assume the causal link between ADHD and anorexia nervosa is firmly established (Levin and Rawana, 2016). Not long ago, it was unlikely to have an ADHD diagnosis and anorexia at the same time because the male:female ratio was 80:20 for ADHD and 3:97 for anorexia nervosa (Bao and Swaab, 2011). That has now changed.

The majority of patients diagnosed with ADHD have difficulty paying attention and controlling their impulses (Willcutt, 2012; Willcutt et al., 2012) and women with eating disorders are now diagnosed with ADHD (Seitz et al., 2013; Stulz et al., 2013; Nazar et al., 2016; Svedlund et al., 2017; Yilmaz et al., 2017). Binge eating in bulimia nervosa is thought to reflect impulsivity, along with a lack of response inhibition (Seitz et al., 2013; Bleck et al., 2015; Pearson et al., 2015) and inattention to the physiology of hunger is thought to play a role in anorexia nervosa (Kaye et al., 2009; Yilmaz et al., 2017).

#### Testing the ADHD Hypothesis

The interview questions and tasks used for estimating and evaluating attention are related mainly to non-food items, including gambling, card sorting and speed of information processing (Rösler et al., 2006; Zastrow et al., 2009; Oltra-Cucarella et al., 2015; Sultson et al., 2016; Tenconi et al., 2016). While these questions may be valid for a diagnosis of ADHD in other subjects, they may be less valid for diagnosing inattention among patients with anorexia nervosa.

Attention is believed to be under top-down control from the prefrontal cortex (Dalley et al., 2011), providing the patients with cognitive control, assisting them in eating only little food. Simultaneously, attenuation of bottom-up control exerted from other brain regions is thought to make the patients inattentive to their starved physiology (Kaye et al., 2009; Brooks et al., 2012a, b). On this framework, one study first noted enhanced activity in the prefrontal cortex in anorexic patients exposed to food items in an fMRI scanner and although bottom-up control was not affected, the patients showed deficiencies in card sorting outside the scanner (Sanders et al., 2015; Sultson et al., 2016). How should these results be interpreted?

As discussed above, the brain has evolved to assist us in searching for food. The activity in the relevant brain neurons outcompetes any activity in neurons engaged in alternative behaviors. Considering that shortage of food, foraging for food, and eating have been the main drivers of evolution (Lieberman, 2011; Ungar, 2017), it would be surprising if someone who had not eaten enough food for a long time, including anorexic patients, was inattentive to food stimuli. Less surprising, a study found that anorexic patients show enhanced attention to food stimuli (Neimeijer et al., 2017), and when examined with a variety of tests of attention, they showed “superior ability to suppress attention to irrelevant information” (Weinbach et al., 2017). Thus, anorexic patients may consider gambling, card sorting and cognitive tests of information processing irrelevant. In fact, activity in their prefrontal cortex may assist them in processing relevant information (Petrovich, 2013; Richard and Berridge, 2013; Ehlers and Todd, 2017).

It is noteworthy that the prefrontal cortex mediates a wide variety of functions, including rational decision making, mentalization, intentionality, and even free will and false beliefs (Denny et al., 2012; Leisman et al., 2012; Funahashi and Andreau, 2013; Mahy et al., 2014; Weele et al., 2018). Even with some compartmentalization within the prefrontal cortex it will be difficult to find a part exclusively devoted to top-down cognitive control of eating.

#### Genetics and the ADHD Hypothesis

It was recently reported that the genetic correlation among ADHD and anorexia is negative (Brainstorm Consortium, 2018; Demontis et al., 2018), but that ADHD and binge eating are genetically related (Capusan et al., 2017). Given this, it has been suggested that the genetic risk factors for ADHD and eating disorders have not yet been clarified (Willsey et al., 2018) and the most recent reviews do not mention eating disorders among the disorders that co-exist with ADHD (Faraone and Larsson, 2019; Glahn et al., 2019).

### Conclusion

The inconsistent outcomes of the many attempts at verifying the mental causation hypothesis have the end result of inadvertently or indirectly supporting Feighner’s hypothesis. Analyses of behavior, cognition and brain function in anorexia nervosa will “make better sense” if they comply with the request for neural plausibility (Crick and Koch, 2003) and if “viewed in the light of evolution” (Dobzhansky, 1973; Jacob, 1977; Striedter et al., 2012). It is surprising that mental disorders, rather that eating behavior, assume priority in most accounts of eating disorders considering that eating behavior has been at the center of animal and human evolution over millennia; chewing is essential for the development of all parts of the head, the brain is not exempt (Lieberman, 2011; Ungar, 2017; Smith, 2018). Keeping this in mind, it seems likely that activities in the brain and other parts of the body assist any starving human, including anorexic patients, in paying attention to food cues. It is suggested that the neglect of the evolutionary perspective rather than methodological and conceptual confusions in neuroimaging research (Donnelly et al., 2018) explains the impasse in the field.

## Validating Feighner’s Hypothesis

*No known medical illness*… *or other psychiatric disorder, that could account for the anorexia* (Feighner et al., 1972)

The present analysis generates the hypothesis that the diagnosis of mental illness, which is valid and reliable for patients with mental disorders, is less suitable for patients with eating disorders. We offer the following retrospective review of clinical records in support of that hypothesis.

### Reliability and Validity of Psychiatric Diagnoses in Eating Disorders

In 2012, 174 patients were admitted to the Mandometer Clinics, most of them through self-referral although some through physician-referral. Following the diagnostic procedures of the clinic (Bergh et al., 2002), 32 were diagnosed with anorexia nervosa, 20 with bulimia nervosa and 122 were diagnosed with an eating disorder not otherwise specified. 37 (21%) of the patients had been referred by a psychiatrist and 20 (54%) of these patients had been diagnosed with a mental disorder, including depression (*n* = 5), anxiety (*n* = 5), and ADHD (*n* = 5).

To examine the reliability and validity of these diagnoses, the Structured Clinical Interview for DSM–IV Axis I Disorders (SCID-I) was administered to all 174 patients by clinical staff trained for the purpose (First et al., 1996; Kaye et al., 2004). 120 (69%) of the patients scored high enough to indicate the existence of a psychiatric diagnosis, with anxiety (43.6%) and depression (31.6%) the most common results of the SCID-I interview. However, there was agreement between the diagnosis made by a psychiatrist and the SCID-I results in only 17/37 (46%) cases, indicating that there was actually disagreement, although this effect was not statistically significant (McNemar test, *p* = 0.26) (Altman, 1990; Table 1). Note that although the SCID-I interview appears sensitive, 26/37 patients (70%) scored high, it failed to confirm the diagnosis made by a psychiatrist in 7/20 (35%) of the cases (Table 1).

**TABLE 1:** Number of patients with a psychiatric diagnosis on admission and number of patients with a psychiatric diagnosis indicated by the SCID-I interview.

		**SCID-I**		
		
		**+**	**−**	**Total**
Psychiatric	+	13	7	20
Diagnosis	−	13	4	17
Total		26	11	37

When the 174 patients were given the opportunity to see a psychiatrist, 60 patients (34%) opted for psychiatric care. However, there was agreement between the outcome of the SCID-I interview and the patient’s subjective experience of a psychiatric problem in only 64 (37%) of the cases (Table 2). There was significant disagreement between the outcome of the SCID-I interview and the patient’s subjective experience of a psychiatric problem (McNemar test, *p* < 0.001) (Altman, 1990).

**TABLE 2:** Number of patients with a psychiatric diagnosis indicated by the SCID-I interview and number of patients taking the chance to visit a psychiatrist.

		**SCID-I**		
		
		**+**	**−**	**Total**
Visit to	+	35	25	60
Psychiatrist	−	85	29	114
Total		120	54	174

### Are Psychiatric Symptoms Among Anorexic Patients Truly Psychiatric Symptoms?

Between 1993 and 2017, 358 patients with anorexia nervosa were treated to remission at the Mandometer Clinics in a median (quartile range) of 18.5 (10–23.2) months. At admission and remission, they completed the Comprehensive Psychopathological Rating Scale (CPRS), that has been demonstrated reliable and valid for estimating OCD, anxiety and depression in patients with these disorders (Svanborg and Åsberg, 1994; Mattila-Evenden et al., 1996; Hanlon et al., 2008).

**FIGURE 1:**
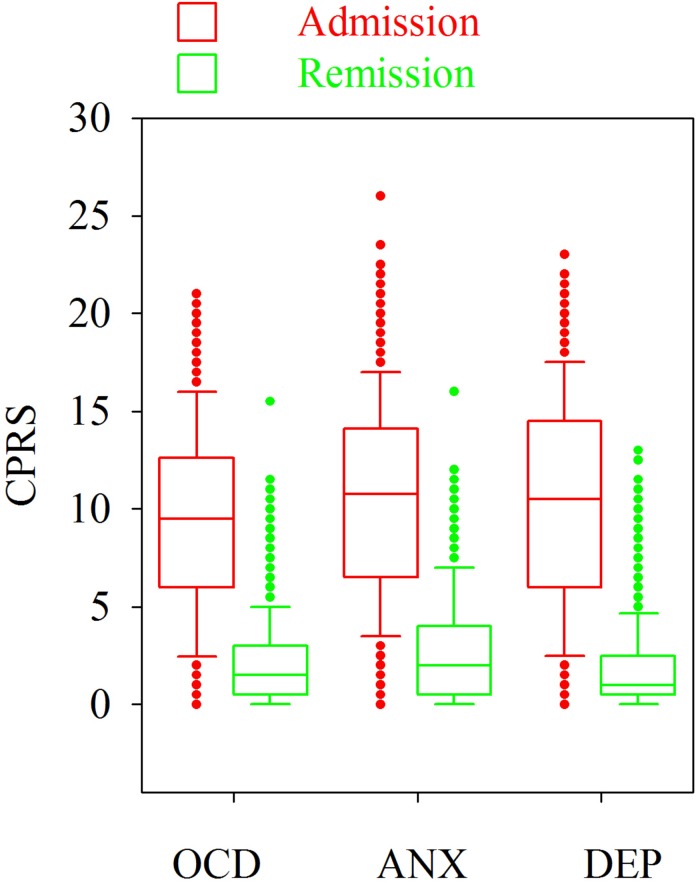
Levels of OCD, anxiety (ANX) and depression (DEP) in 358 patients with anorexia nervosa at admission and at remission determined with the Comprehensive Psychopathological Rating Scale (CPRS).

**FIGURE 2:**
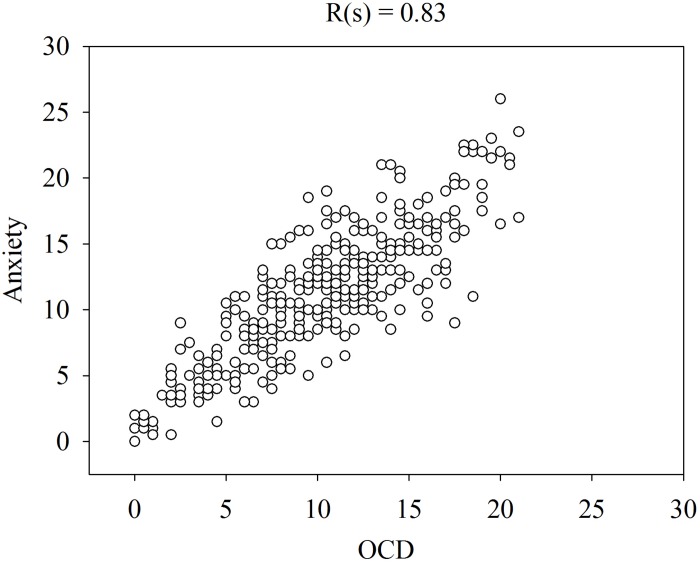
Spearman rank correlation [R(s)] among anxiety and OCD in 358 patients with anorexia nervosa determined with the CPRS.

**FIGURE 3:**
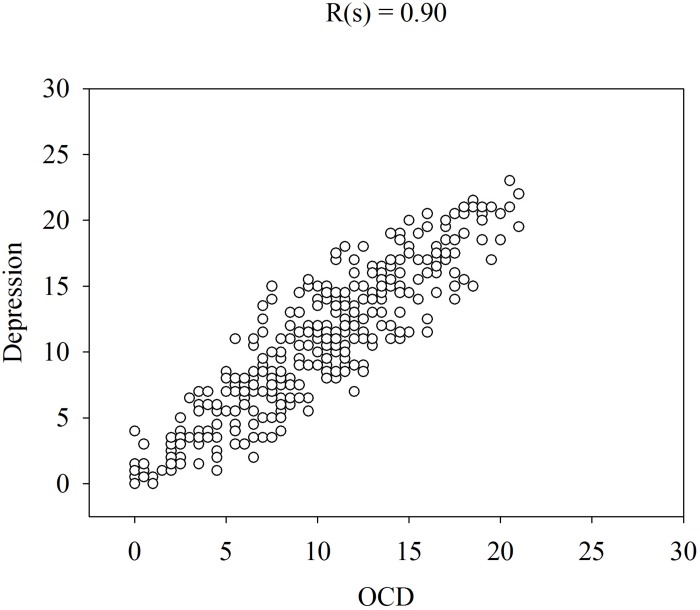
Spearman rank correlation [R(s)] among depression and OCD in 358 patients with anorexia nervosa determined with the CPRS.

**FIGURE 4:**
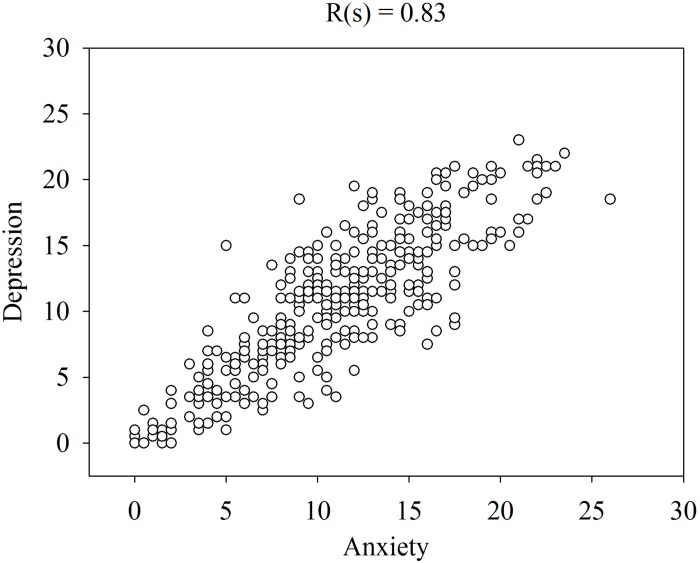
Spearman rank correlation [R(s)] among depression and anxiety in 358 patients with anorexia nervosa determined with the CPRS.

Figure 1 shows that the patients rated the level of these symptoms high at admission and low at remission. Please note that the levels of OCD, anxiety and depression were similar on both occasions and that some patients rated their symptoms low at admission and some rated their symptoms high at remission. The similarities in the ratings of OCD, anxiety and depression are verified by the high correlations (Spearman rank correlation) between these symptoms at admission (Figures 2–4).

**FIGURE 5:**
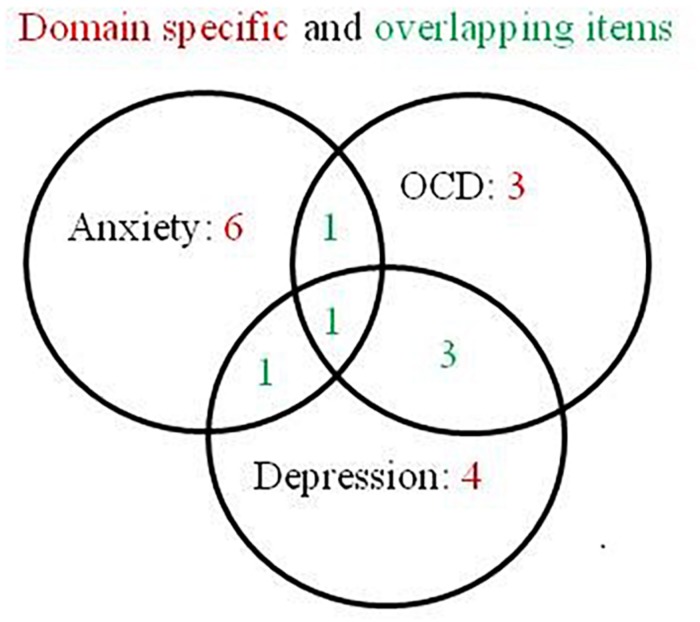
Domain specific and overlapping items for anxiety, OCD and depression in the 19 items of the Comprehensive Psychopathological Rating Scale.

The CPRS as used for the diagnosis of eating disorders includes 19 items, some of which are specific for OCD, anxiety and depression and some are overlapping between these three domains (Figure 5). A nonparametric Mokken scalability analysis (Sijtsma and Molenaar, 2002) showed that all items contributed to just one underlying variable (all scalabilities except one were >0.4). One item (scalability = 0.2) indicated more noice than information. A good contributing item should show a scalability >0.3. The result is not surprising as there is a set of items thought to contribute to more than one domain. A subsequent automated item selection procedure (Sijtsma and Molenaar, 2002) with the domain overlapping items excluded, should reveal the three intended domains, but the analysis confirmed the first finding of just one underlying domain, with no evidence of any underlying secondary domain. A parametric Rasch model (Sijtsma and Molenaar, 2002) yielded the same result, one dominating domain with high person – item reliabilities of 0.91 and 0.98, and with two items indicating more noice than information. A principal component analysis of the standardized residuals could not find any additional domain (1st and 2nd contrast <2).

It is noteworthy that at admission, 78 of the patients had been prescribed at least one psychoactive drug, 46 were given an antidepressant, 24 were given an anxiolytic, and 25 were given a neuroleptic, alone or in combination. Their time to remission was 16.7 (10.4–16) months. In our clinics, psychoactive drugs are gradually reduced as much as possible as part of our treatment, and at remission 7 patients were still taking an antidepressant, 7 were taking an anxiolytic, and 12 were taking a neuroleptic. The CPRS ratings for patients taking psychoactive drugs were as high as for the other patients at admission, and as low as the other patients at remission (data not shown separately), indicating that they appeared to make no difference in the outcomes either of the treatment for eating disorders, or in the outcomes for their psychiatric symptoms.

### Discussion and Conclusion

These data bring into question whether the diagnosis of psychiatric symptoms, using the procedures that have been proven effective for patients with psychiatric disorders (Drill et al., 2015; Huprich et al., 2015; Hutsebaut et al., 2016) are helpful in treating patients with anorexia nervosa. The domain-specific items of the CPRS failed to dissociate OCD, anxiety and depression in 358 anorexic patients and instead pointed to one domain, thereby raising the question about what the underlying variable might be. Could it be that the single issue that triggers these symptoms is starvation? It is tempting to speculate that the symptoms assessed by the CPRS do not reflect typical symptoms for OCD, anxiety or depression that is seen in anorexic patients. Indeed, the ineffectiveness of psychoactive drugs targeting these symptoms in anorexic patients is well known (Fineberg et al., 2015). It might be significant in this context that mental symptoms associated with eating disorders are inconsistent over time, identifying some of these symptoms at any particular time may be difficult, as they may be remitting and relapsing over time, rather than running a chronic course (Anckarsäter et al., 2012). Equally significant, by practicing how to eat normally, patients with anorexia nervosa and other eating disorders as well remit from psychiatric symptoms when psychoactive drugs prescribed for treating the same symptoms are withdrawn (Bergh et al., 2002, 2013).

It is concluded that the mental symptoms are effects of eating disorders.

## Summary

Very few patients with anxiety develop anorexia nervosa but it is common that starving anorexia patients exhibit anxiety. The risk of developing anorexia nervosa among patients with mental disorders has been overestimated by calculating relative ratios among low probabilities. Reports on inattention in patients with eating disorders are based on tests that may be irrelevant for someone who is starving. When relevant tests are used, anorexic patients show increased attention. There are no convincing data relating the genetics of psychiatric disorders with the genetics of anorexia. All the failed attempts at supporting the mental causation hypothesis, including its subsidiary genetic hypothesis, can be interpreted as supporting Feighner’s hypothesis that the starvation of anorexia causes its mental symptoms.

A word of caution seems appropriate. Thus, most of the literature reviewed is descriptive, putting constraints on cause-effect analyses (Pearl, 2019). While some experiments have tested the hypothesis that cognitive processes maintain the psychopathology of patients with eating disorders, the results are inconclusive (Södersten et al., 2017). Yet, it remains possible that a psychopathology turns out to be a cause of anorexia.

Psychiatric diagnostic procedures appear to be better suited for patients with psychiatric disorders than for patients with anorexia nervosa and perhaps other eating disorders as well. If psychiatric illness does not cause eating disorders we need to consider alternatives such as that starvation causes anxiety, depression and obsession. If this is the case, then we can assume that starvation is the critical problem that needs to be addressed. If we look at this as a behavioral issue and take an evolutionary perspective to understanding the behavior, we can develop treatments that may target the problem. For this purpose a theoretical framework is useful.

## Framework

*A simple, parsimonious hypothesis is that self-starvation is initially rewarding and subsequently controlled by conditioning to previously neutral stimuli* (Bergh and Södersten, 1996)

Feighner et al. (1972) noted that anorexic patients are physically hyperactive and that they are rewarded for maintaining a low body weight. In 1996, Feighner’s hypothesis was formalized, relating the patients’ behavioral phenotype to the brain mechanisms of reward and attention (Bergh and Södersten, 1996). In the context of the present Research Topic, and in the spirit of Occam’s razor, i.e., leaving what is unimportant in the standards of care for eating disorders without notice (Gutiérrez and Carrera, 2018), we would like to re-launch the 23 year old hypothesis cited in the quote. It is actually a framework rather than a hypothesis. Crick and Koch have explained what the term “framework” means in an analysis that we have adapted to understanding eating disorders (Crick and Koch, 2003; Zandian et al., 2007). For example, a framework should be plausible relative to available evolutionary and neurobiological data (Crick and Koch, 2003).

It is helpful to consult Guisinger once more. Thus, she stressed the distinction between proximate (physiological) and ultimate (evolutionary) causes of anorexia (Guisinger, 2003). On the present framework, the ultimate cause of anorexia is the lack of food, that has plagued human evolution, and the proximate cause is the associated neuroendocrinology of reward and attention and of foraging for food. However, “proximate cause” equals “mechanism” and mechanisms are distinct from causes as noted in the last paragraph of section “Co-morbidity.”

Today, women (and some men as well) diet to avoid becoming overweight and Guisinger suggested that “psychological factors” might make some individuals more prone to dieting than others (Guisinger, 2003). However, one of the best studied of these psychological factors, restrained eating, is similar to the mental symptoms discussed in this overview in that it is more likely an effect than a cause of disordered eating behavior (Zandian et al., 2009; Johnson et al., 2012).

On the hypothesis that eating disorders are behavioral issues that should be understood on an evolutionary perspective, our framework has been translated into clinical practice over 25 years, the theoretical basis, the treatment, and its outcomes have been described in detail elsewhere (Bergh et al., 2002, 2013, Södersten et al., 2008, 2014, 2017, 2019a). In brief, a reduction in food intake causes release of dopamine in the forebrain terminals of the mesolimbic dopamine neurons concerned with reward, encouraging the patient to continue dieting (Bergh and Södersten, 1996). Hence, dieting “causes” anorexia nervosa via dopamine release in the ventral striatal terminals of the neural network of “reward.” The hypothesized rewarding role of dopamine has now been confirmed (Södersten et al., 2016). In addition, it was originally hypothesized that because dieting also activates the brainstem noradrenalin neurons concerned with attention, anorexia is maintained by conditioning to the situations which provide reward (Bergh and Södersten, 1996). This long established line of research has been confirmed and extended in detail over the years (*ibib*).

The main intervention, teaching patients how to eat using real time visual feedback on how to eat and how much food to eat during the meal, was recently published in a video (Esfandiari et al., 2018). In addition, the mechanism whereby eating behavior affects mental symptoms (although the nature of these symptoms needs to be determined), has been outlined engaging brainstem relays for chewing as parts of a neural network that connects to limbic forebrain areas, including the prefrontal and orbitofrontal cortex (Ioakimidis et al., 2011).

## Hypothesis

*You are how you eat* (Zandian, 2009; Lieberman, 2011)

Chewing is at the heart of human evolution, promoting brain development and many aspects of physical and mental health (Lieberman, 2011; Kumar et al., 2018). In addition, chewing exerts an anxiolytic effect (Hollingworth, 1939; Ioakimidis et al., 2011). Thermoregulation also played a key role in evolution (Jastroch et al., 2018), warmth promotes health (Heinonen and Laukkanen, 2018) and recovery from activity-based anorexia in experimental animals (Carrera and Gutiérrez, 2018). In addition, warmth exerts an anxiolytic effect in anorexic patients (Zandian et al., 2017) and warmth has been used to treat anorexic patients over 145 years (Gull, 1874; Bergh et al., 2002; Carrera and Gutiérrez, 2018).

Both chewing and warmth exert their anxiolytic effect by engaging the serotonin neurons in the dorsal raphe nucleus in the brainstem that project to the prefrontal and orbitofrontal cortex (Lowry et al., 2009; Ioakimidis et al., 2011). These limbic brain regions are influenced by chewing within 30 min (Svensson et al., 2006), the time it takes for warmth to reduce anxiety (Zandian et al., 2017). This limbic cortical plasticity may extend to striatal dopamine neurons, which are also essential for mental health (Kita and Kita, 2011). The hypothesis emerges that chewing and warmth exert their anxiolytic effect by engaging the prefrontal cortex.

### Testing the Hypothesis

Transcortical magnetic stimulation and recording of evoked motor potentials in peripheral muscles (Kumar et al., 2018) combined with chewing and standard methods for examining anxiety and administering heat in the clinic (Zandian et al., 2017) make testing of the hypothesis feasible.

## Conclusion

… *the underlying assumption that* [*anorexia nervosa*] *is always caused by disturbed psychological processes may not always be true* (This Research Topic)

The mental causation hypothesis, formalized as the OCD- and ADHD-hypothesis, has not been supported possibly because mental disorders vary along continuous dimensions and cannot be divided into discrete categories. This was realized 100 years ago (Fisher, 1918) and is now re-realized (Wu, 2008; Insel et al., 2010; Adam, 2013; Gillan et al., 2017; Brainstorm Consortium, 2018).

Biological hypotheses make sense only if viewed in the light of evolution, the mental causation hypothesis cannot be supported when examined from an evolutionary perspective. The commonly held assumption that individuals with anorexia do not pay attention to their physiological signals is undermined when one takes into account the broader impact of starvation. When appropriate stimuli are used, individuals with anorexia pay more, rather than less, attention to hunger cues. Starvation was the major evolutionary threat and humans have evolved to eat large meals whenever offered the chance, not as a result of lack of response inhibition. The high level of physical activity, the difficulty in obtaining food, and the constant low body weight exemplify the meaning of the term “homeostasis” as this concept emerged from the ideas of Bernard subsequently tested by Cannon (Södersten et al., 2006, 2008, 2011).

Interestingly, it was recently suggested that the genes related to anxiety were adaptively selected for at a crucial period of human development, during human migration “Out-of-Africa” about 100, 000 years ago, and have been maintained in the population (Sato and Kawata, 2018). Furthermore, the same genotype may have offered reproductive advantage over the course of evolution (Alvergne et al., 2010).

We have pointed out the inconsistencies in the mental causation hypothesis continuously since 1996 and in line with the present Research Topic we have offered an alternative framework and translated it into clinical practice over the years. The main intervention of our treatment is to teach patients to eat normally, because “eating disorders are eating disorders,” not mental disorders. As noted repeatedly in the present account, eating behavior, chewing in particular, has been of major importance in evolution (Lieberman, 2011; Ungar, 2017; Smith, 2018), and it is unsurprising that disordered eating is associated with health problems. Interestingly, this framework has been extended to the general hypothesis that inappropriate use of our muscles is a major cause of several health problems (Lieberman, 2014).

The hypothesis offered here is but one of several possible hypotheses that emerges from our framework and aims at establishing the link among eating behavior, the brain, and psychological epiphenomena. Obviously, another question comes to mind: if psychiatric symptoms in patients with mental disorders are different from the “psychiatric symptoms” of anorexic patients, what is the dimension(s) underlying these symptoms in anorexia nervosa?

## Personal Insights and Opinions

Normalization of eating behavior was demonstrated to be effective in a pragmatic randomized controlled trial (Bergh et al., 2002) and treatment outcomes were subsequently reported for 1,428 patients, including all eating disorders diagnoses, from six clinics in four countries (Bergh et al., 2013). The outcomes of this treatment was then shown to be better than the outcomes of cognitive behavioral therapy for the treatment of eating disorders, the treatment that is thought to rest on the best evidence (Södersten et al., 2017). A recent analysis of the data in the National Quality Registry for the Treatment of Eating Disorders in Sweden found that outcomes of this eating-normalization treatment has been better over several years than the outcomes at the other clinics in Sweden (Södersten et al., 2019a). The differences in outcomes were not the result of a difference in patient characteristics at admission. In fact, our patients are more severely ill than patients admitted to other clinics (Södersten et al., 2016).

The success of our treatment in comparison to that in the published literature supports the tenets upon which it is built/was developed. Clearly, it is time to reconsider the mental causality hypothesis and consider eating behavior and its underlying evolutionary adaptiveness. Once we focus on the effects of starvation on eating behavior, the treatment of anorexia and other eating disorders can be improved. Interestingly, the first step in cognitive behavioral therapy for eating disorders is similar in that “*the prescription of a pattern of regular eating* (italics in original) is probably the single most effective procedure in the treatment,” which “in the great majority of cases results in a marked … decrease in the level of general psychiatric symptoms” (Fairburn et al., 1993).

## Ethics Statement

The analysis of the data in the clinical files, i.e., the registry of the Mandometer Clinics (point 4), was approved by the Regional Ethical Review Board of Stockholm. The data has been collected since 1993 and is continuously collected. The patients in the registry did not participate in the analysis. All patients entering treatment are informed verbally and in writing that their data might be used in research and if their data are used in research it will be anonymized. Written consent by the patients is not required for analysis of data collected in registries over long periods of time. The patients are also informed that they can request that their data is not used and that they can leave the treatment any time without giving a reason.

## Author Contributions

PS, MZ, and CB collected the data. UB performed the statistical analyses. CB supervised the clinical work since 1993. All authors conceived the idea of the manuscript, analyzed the data, reviewed the several versions of the manuscript, and approved the final version of the manuscript. PS wrote the manuscript.

## Conflict of Interest Statement

The authors declare that the research was conducted in the absence of any commercial or financial relationships that could be construed as a potential conflict of interest.
